# The association between symptoms of mental disorders and health risk behaviours in Vietnamese HIV positive outpatients: a cross-sectional study

**DOI:** 10.1186/s12889-017-4162-6

**Published:** 2017-03-14

**Authors:** Truc T. Thai, Mairwen K. Jones, Lynne M. Harris, Robert C. Heard

**Affiliations:** 1 Faculty of Public Health, Ho Chi Minh City University of Medicine and Pharmacy, 159 Hung Phu Street, Ward 8, District 8, Ho Chi Minh City, Vietnam; 2 Faculty of Health Sciences, University of Sydney, 75 East Street, Lidcombe, Sydney, NSW 2141 Australia; 3 School of Psychological Sciences, Australian College of Applied Psychology, Level 11, 255 Elizabeth Street, Sydney, NSW 2000 Australia

**Keywords:** Mental health disorder, Health risk behaviours, HIV/AIDS, Outpatient, Vietnam

## Abstract

**Background:**

A high prevalence of symptoms of mental disorders (SOMD) has been found among people living with HIV/AIDS (PLHIV). Additionally, SOMD may impact on the prevalence of high-risk health behaviours (HRB). This study investigates the relationship between SOMD and HRB in a large sample of Vietnamese HIV positive outpatients.

**Methods:**

A cross-sectional study was conducted with 400 outpatients at two HIV/AIDS clinics in Ho Chi Minh City, Vietnam, selected using a systematic sampling technique. Validated scales were used to measure SOMD, specifically symptoms of depression, anxiety, alcohol use disorder (AUD), substance use disorder (SUD) and HIV associated dementia (HAD). Participants completed a self-report questionnaire assessing HRB during the preceding 12 months including unsafe sexual practices and illicit drug use. Multivariable logistic regression models were used to evaluate associations between SOMD and HRB.

**Results:**

The majority of participants (63.5%) were male and the median age was 34.0 years. Unsafe sexual practices and illicit drug use were reported by 13.8 and 5.5% of participants. The prevalences of HAD, depression, AUD, anxiety and SUD symptoms were 39.8, 36.5, 13.3 10.5, 3.3% respectively. There was no association between SOMD and HRB either with or without adjusting for correlates of HRB, except between symptoms of SUD and illicit drug use. PLHIV who had symptoms of SUD were more likely to use illicit drugs (adjusted Odds Ratio 81.14, 95% CI 12.55–524.47).

**Conclusions:**

While the prevalence of SOMD among HIV positive outpatients was high, most SOMD were not associated with increased HRB. Only illicit drug use was predicted by symptoms of SUD. Screening PLHIV for symptoms of SUD may be useful for detecting people likely to be engaging in illicit drug use to reduce the risk of secondary disease transmission.

## Background

People living with HIV/AIDS (PLHIV) who are aware of their HIV-positive status may continue to engage in high-risk health behaviours (HRB) [1–3]. Unsafe sexual practices (USP) and injecting drug use among PLHIV represent important sources of secondary disease transmission [4, 5]. Studies conducted in Russia and America have found that up to 50% of PLHIV reported having had unprotected vaginal or anal intercourse with sero-discordant partners [3, 5–7]. In Vietnam, about 20% of PLHIV reported having sex with multiple partners and less than half said that they consistently used condoms with regular or casual sexual partners [2]. Such HRB may result in HIV transmission to HIV-negative persons. Additionally, PLHIV engaging in these HRB are more likely to become infected with new HIV strains or other sexually transmitted diseases which can lead to acceleration of HIV, HIV treatment resistance, or secondary HIV transmission [5, 8, 9].

Substance use, regardless of the intake method, has been found to be common in PLHIV. In a sample from Russia, about 35% of PLHIV reported injecting drug use and 63% of this group indicated that they also shared needles [3]. In Vietnam, slightly more than half (51.6%) of a sample of 4266 PLHIV reported injecting drug use during the past month and 35% of these reported that they had shared needles [2]. A review of 12 studies undertaken in Europe and one study conducted in the USA revealed a prevalence of recreational substance use in PLHIV of approximately 30% [10, 11]. Substance use has been shown to complicate HIV treatment since the acute effects of intoxication lead to difficulties with medication adherence, which may result in accelerating HIV disease progression [12, 13]. Overall, USP, injecting drug use and other substance use can increase the likelihood that individuals will be exposed to a range of diseases such as hepatitis B and C, herpes, syphilis, chlamydia and gonorrhoea [14, 15], further compromising the health of PLHIV. As such, research that elucidates the contributors to HRB is crucial for intervention and prevention programs.

The relationship between symptoms of mental disorders (SOMD) and HRB among PLHIV is multifaceted and complex [4]. PLHIV may experience depression or anxiety after being informed that they are HIV positive due to fear of developing AIDS, or being aware of the stigma and discrimination towards HIV/AIDS [4, 16]. Also, PLHIV who have SOMD may be at greater risk of engaging in HRB due to impaired judgment and reduced ability to accurately assess or negotiate risk [5–7]. For example, in a sample of 360 American PLHIV it was found that those who were severely depressed, socially anxious or cognitively impaired were less able to negotiate condom use [7]. Further, HRB such as substance use among PLHIV can result in manic or psychotic episodes during which the person experiences impaired judgment [17]. Finally, substance dependence can lead to HRB, such as sharing drug use equipment or engaging in unprotected sex when intoxicated, and this can result in secondary HIV infection [4, 16].

A meta-analysis of 34 studies conducted prior to 2000 showed little evidence to support a relationship between SOMD, HRB and presence of HIV [18]. However, this meta-analysis was criticized for methodological inadequacies including that cross-sectional studies pooled in the analysis could not demonstrate a temporal association between SOMD and HRB [19]. Several recent investigations have found a significant, positive association between SOMD and HRB in PLHIV [6, 20, 21]. For example, a longitudinal study among 851 American PLHIV demonstrated that those with symptoms of depression and anxiety engaged in increased risky sexual behaviours and substance use [20]. However, other research has found that depression and anxiety among PLHIV may reduce sexual desire, pleasure and frequency of sexual activities [22], thus resulting in a negative association between SOMD and HRB. Given the somewhat contradictory findings concerning the relationship between SOMD and HRB among PLHIV further research is required. Understanding this relationship is crucial since SOMD such as symptoms of depression, anxiety and substance use are treatable, so addressing SOMD may assist in limiting HRB among PLHIV and thus improve secondary HIV prevention [23].

Numerous factors are associated with USP, injecting drug use and other substance use among PLHIV worldwide. Predictors of USP in a sample of Indian PLHIV included being female, childless, and knowledge that partner was HIV positive [24], and predictors in American PLHIV included younger age, and absence of AIDS complications [5]. In a sample of Vietnamese PLHIV predictors of USP included lower educational attainment and higher number of sexual partners [2]. Variables not previously found to be associated with USP include ethnicity, time since HIV diagnosis, CD4 cell count, viral load [5], general health status, tuberculosis co-infection [24] and presence of other sexually transmitted diseases [3]. The predictors of illicit drug use are identical to those identified for USP, with the addition of drug dependence, higher number of HIV symptoms [25], lower level of HIV treatment adherence in American PLHIV [26], and being informed of one’s HIV status in Romanian PLHIV [27]. Identifying the predictors of HRB can assist in identifying PLHIV who are most at risk of undertaking HRB and thus developing targeted strategies to reduce HRB among those at high risk, therefore leading to better health outcomes and quality of life for PLHIV in Vietnam.

In 2015, Vietnam had approximately 254,000 PLHIV with 50.8% reporting that their HIV infection had occurred through unprotected sex and 36.1% reporting transmission through blood contact including injecting drug use. Ho Chi Minh City (HCMC) is the centre of economic activity in Vietnam and is also recognised as having the highest number of PLHIV in Vietnam, with 49,561 PLHIV, nearly 20% of the national total in 2015 [28]. Of these, about 25,000 are registered and receive treatment at 30 HIV/AIDS outpatient clinics, mostly located at district health care centres [28]. Each clinic employs the same national guidelines for the admission and management of clients. Currently, the people most at risk of becoming infected with HIV in Vietnam are those who inject drugs, with 9.3% of this group HIV positive, compared to 2.7% of female sex workers and 5.2% of men who have sex with men [28]. While researchers have investigated factors associated with HRB among Vietnamese PLHIV including knowledge and beliefs about HIV, disclosure of HIV status, partner characteristics, social support, HIV treatment and health status [2, 22, 29], there is a paucity of research investigating the relationship between SOMD and HRB in this population in Vietnam. With prevalence of depression in Vietnam estimated to be up to 40% based on screening scales [30] and 31% using psychiatric interview [31] and given evidence that SOMD are associated with HRB in a large national sample (*N* = 10,783) in the USA [32], research examining the relationship between SOMD and HRB among PLHIV in Vietnam is warranted.

This study investigated the relationship between a range of frequently occurring SOMD, specifically symptoms of depression, anxiety, alcohol use disorder (AUD), substance use disorder (SUD) and HIV associated dementia (HAD), and their relationship with USP and illicit drug use in a large sample of Vietnamese HIV positive outpatients in HCMC.

## Methods

### Setting and participants

We conducted a cross-sectional study from December 2013 to March 2014 at two HIV/AIDS clinics randomly selected from the 30 public HIV/AIDS outpatient clinics in HCMC. These two clinics were selected by placing 30 envelopes each with one clinic’s name in a box and randomly drawing two of the envelopes. The study was confined to two clinics due to the limitation of study resources. The study was part of a larger research project that involved all participants being interviewed by a psychiatrist and all participants being followed-up 3 months after the initial assessment. As the study required all participants to be interviewed and followed up, it was not feasible to include additional HIV clinics in the time available for the study. Details of the procedures and results from psychiatrists’ interviews have been reported elsewhere [31]. The two HIV clinics were situated within district health care centers located in District 8 and District 10. These districts are two of 19 inner urban districts of HCMC. District 8 is in Southeast HCMC, comprises 16 wards and has a population of more than 420,000 people. District 10 is in Southwest HCMC, comprises 15 wards and has a population of more than 235,000 people. Each HIV clinic provides services for HIV positive outpatients who reside in the district. At any time, the HIV clinic in District 8 has about 1400 patients while the HIV clinic in District 10 has about 1300 patients, and most patients at both HIV clinics will be receiving ART. Both district health care centers where the HIV clinics are located also include departments of mental health that deliver mental health services. However, there is no formal linkage between the HIV clinics and the mental health services and no routine screening for SOMD at the HIV clinics for referral to mental health services.

The formula and parameters used for the sample size calculation were based on those used for a larger study to estimate prevalence of HIV positive outpatients who had SOMD requiring a referral to psychiatric services. In summary, with expected prevalence of 0.50 and the width of 95% confidence interval of 0.10, at least 385 outpatients were needed [31]. With prevalence of HRB among outpatients with and without SOMD of 0.70 and 0.20, a sample of at least 18 outpatients with SOMD and 18 outpatients without SOMD was needed to detect relationships of moderate strength with at least 80% power [31]. Therefore, a rounded number of 400 was sought, taking into account participants lost to follow up in the larger research project. Inclusion criteria were as follows: (a) at least 18 years of age; (b) sufficient literacy to respond to the questionnaire; and (c) commenced ART treatment at least 30 days previously. This final inclusion criterion was important because side-effects of the ART medication which affect physical and/or mental health are most likely to occur when ART is first initiated, so that including those who had commenced ART within 30 days may have resulted in an overestimate of the prevalence of SOMD in this population. Since a sample of 200 outpatients was sought from each HIV clinic, every fifth outpatient who met the inclusion criteria was systematically recruited on the day of their routine monthly visit based on the assigned ticket number when the patient arrived at the clinics. A total of 400 HIV positive outpatients, 200 from each clinic, agreed to participate from the 410 HIV positive outpatients initially targeted for recruitment.

### Procedure and measurement

#### Self-report questionnaire

Participants completed a self-administered questionnaire that asked about demographic characteristics, general health status, HIV status disclosure, practical, emotional, spiritual and financial support occasionally or frequently received from family, society and professionals, and number of stressful life experiences during the last 30 days. The questions about stressful life experiences were adapted from previous measures of HIV-related stress [33–35].

#### Clinical data extraction

The researcher (first author), with the assistance of staff at the clinics, extracted health and treatment-related data from the clinical records including weight (kg), height (m), time since HIV diagnosis (years), time since ARV treatment (years), most recent CD4 cell count (cells/mm^3^), HIV stage (1, 2, 3, 4), presence of other chronic diseases (Yes/No), history of delaying ARV treatment (Yes/No), history of opportunistic disease (Yes/No), adherence to HIV medications (Good/Average/Poor) and whether family members had been diagnosed HIV positive (Yes/No). Body mass index was calculated from weight and height and was categorized based on an Asian population. Viral load was not extracted as it is only assessed for patients with indicators of treatment failure in Vietnam and was not available for the majority of these outpatients.

#### Symptoms of mental disorders

Symptoms of depression were measured using the Center for Epidemiologic Studies–Depression scale (CES-D) [36]. The CES-D has 20 items and the total score ranges from 0 to 60. Cutoffs of 16, 21 and 25 have been used in previous studies to screen for depression [37] but the cutoff of 16 was used here as this has been widely used in research and recommended by the developer to indicate likelihood of clinical symptoms of depression during the past 7 days [36]. Anxiety symptoms were evaluated using the 13 item Phan Vietnamese Psychiatric Scale-Anxiety subscale (PVPS-A) [38]. Based on previous work [38] a mean score of greater than 1.60 on the PVPS-A was used to indicate probable anxiety disorder during the last 4 weeks. Symptoms of SUD were assessed using the 10-item Drug Abuse Screening Test (DAST) [39]. Cutoffs of 1, 3, 6 and 9 have been used in previous studies [39, 40] however the cutoff of 3 has been commonly used in studies that include people with and without drug use problems [40, 41] and therefore a total score of 3 or more on the DAST was used to indicate clinical symptoms of SUD during the past 12 months. Symptoms of AUD were measured by the World Health Organization Alcohol Use Disorder Identification Test (WHO-AUDIT) [42]. Cutoffs of 8, 16 and 20 have previously been used to screen for symptoms of AUD on the WHO-AUDIT [42] and we used the cutoff of 8, as this is the most commonly reported cutoff to indicate symptoms of AUD during the last 12 months both in the general population and among PLHIV [42–44]. Symptoms of HIV associated dementia (HAD) were measured using the International HIV Dementia Scale (IHDS) [45]. A score of ≤ 10 on the IHDS has been recommended for detecting current HIV associated dementia among PLHIV [45] and a cutoff of 10 was used in the present study. These scales have been shown to have good psychometric properties and have been used in previous studies with PLHIV [36, 38, 41, 42, 45, 46].

#### High-risk health behaviours

HRB was assessed by self-reported USP and illicit drug use. Illicit drug use was indicated when participants reported injecting drug or using other recreational drugs during the past 12 months. USP were identified if the participants responded that they had occasionally, frequently or very frequently had anal intercourse or vaginal intercourse with their partners or with sex workers without protection in the past 12 months.

### Data analysis

Proportions for categorical variables and medians with inter-quartile ranges (IQR) for continuous variables were used to summarize the data. To detect the differences among those with and without USP or illicit drug use, we used either the Chi-squared tests or the Fisher exact tests for categorical variables and the Wilcoxon Rank Sum tests for continuous variables. Simple logistic regression models were fitted to evaluate the association between independent variables and USP or illicit drug use. Multivariable logistic regression was carried out to identify correlates of each of the outcome variables using the procedures recommended by Hosmer et al. [47]. First, independent variables with a *p*-value of ≤ 0.2 in bivariable analyses were used in the initial multivariable logistic regression model. Then, variable retention in the next models was based on a *p*-value <0.05 from the initial model. Each non-significant variable from the initial model was reintroduced to assess its individual contribution to the model. Variables that contributed to a change of >20% of estimates and the interaction such as between sex, age with SOMD were also investigated before fitting the final model. The association between SOMD and HRB was analysed using multivariable logistic regression adjusting for correlates identified in the above procedure. All analyses were carried out using Stata v13 (StataCorp, College Station, TX).

### Ethics

The study was reviewed and approved by the Human Research Ethics Committee of the University of Sydney, Australia (2013/859) and the HCMC Provincial AIDS Committee, Vietnam (IRB-03-2013). All participants provided signed informed consent.

## Results

### Participants’ characteristics

The majority of participants were male (64%) and the median age was 34.0 (IQR 30.5–38.0) years. The median time since HIV diagnosis was 5.7 (IQR 3.2–6.7) years. Almost all participants (99.0%) were on ART and the median number of years on treatment was 4.2 (IQR 1.8–5.9). Unsafe sexual practices and illicit drug use were reported by 13.8 and 5.5% of participants respectively. Over one third of respondents were classified with symptoms of HAD (39.8%) and depression (36.5%), followed by symptoms of AUD (13.3%), anxiety (10.5%), and SUD (3.3%).

### Bivariable analysis

Unsafe sexual practices were associated with younger age (OR = 0.95, 95% CI 0.90–0.99) and higher education level (OR = 2.65, 95% CI 1.20–6.03), higher economic status (OR = 2.27, 95% CI 1.22–4.35) (Table 1), poorer adherence to HIV medication (OR = 3.62, 95% CI 1.16–11.24) (Table 2), having a family member diagnosed HIV positive (OR = 2.13, 95% CI 1.19–3.81), receiving social support from HIV support networks (OR = 2.00, 95% CI 1.08–3.70) and not having professional support (OR = 1.82, 95% CI 1.02–3.23) (Table 3). However, USP were not associated with symptoms of depression (OR = 0.91, 95% CI 0.50–1.65), anxiety (OR = 1.29, 95% CI 0.54–3.07), AUD (OR = 0.95, 95% CI 0.40–2.22), SUD (OR = 1.15, 95% CI 0.25–5.31) or HAD (OR = 1.01, 95% CI 0.57–1.81).

**Table 1:** Bivariable association between health risk behaviours and socio-demographic characteristics among Vietnamese HIV positive outpatients (*N* = 400)

Demographic	Unsafe sexual practices				Illicit drug use			
	Yes *n (%)*	No *n (%)*	*p*	OR (95% CI)	Yes *n (%)*	No *n (%)*	*p*	OR (95% CI)
Sex								
Female	25 (17.1)	121 (82.9)	0.137	1	2 (1.4)	144 (98.6)	0.005	1
Male	30 (11.8)	224 (88.2)		0.65 (0.36–1.15)	20 (7.9)	234 (92.1)		6.25 (1.41–25.00)
Sexual orientation								
Heterosexual	51 (14.5)	300 (85.5)	0.370	1	18 (5.1)	333 (94.9)	0.273	1
Homosexual/Bisexual	3 (13.0)	20 (87.0)		0.88 (0.25–3.08)	1 (4.3)	22 (95.7)		0.84 (0.11–6.59)
Unsure/Not answered	1 (3.8)	25 (96.2)		0.24 (0.03–1.77)	3 (11.5)	23 (88.5)		2.41 (0.66–8.79)
Age in years								
≤ 30	21 (21.0)	79 (79.0)	0.035	1	7 (7.0)	93 (93.0)	0.642	1
31–40	29 (12.2)	208 (87.8)		0.52 (0.28–0.97)	13 (5.5)	224 (94.5)		0.77 (0.30–1.99)
≥ 40	5 (7.9)	58 (92.1)		0.32 (0.12–0.91)	2 (3.2)	61 (96.8)		0.44 (0.09–2.17)
Median (IQR)	31 (30–37)	34 (31–38)	0.022	0.95 (0.90–0.99)	33.5 (29–36)	34 (31–38)	0.526	0.98 (0.91–1.05)
Work status								
Unemployed/Casual	21 (13.8)	131 (86.2)	0.893	1	8 (5.3)	144 (94.7)	0.862	1
Part-time	9 (13.0)	60 (87.0)		0.94 (0.40–2.16)	4 (5.8)	65 (94.2)		1.11 (0.32–3.81)
Full-time	16 (12.7)	110 (87.3)		0.91 (0.45–1.82)	6 (4.8)	120 (95.2)		0.90 (0.30–2.67)
Not working	9 (17.0)	44 (83.0)		1.28 (0.54–2.99)	4 (7.5)	49 (92.5)		1.47 (0.42–5.09)
Highest level of education completed								
≤ Primary school	20 (14.4)	119 (85.6)	0.030	1 ^a^	8 (5.8)	131 (94.2)	0.873	1
Secondary school	13 (8.6)	138 (91.4)		0.56 (0.27–1.17)	9 (6.0)	142 (94.0)		1.04 (0.39–2.77)
≥ High school	22 (20.0)	88 (80.0)		1.49 (0.76–2.89)	5 (4.5)	105 (95.5)		0.78 (0.25–2.45)
Marital status								
Single	14 (11.3)	110 (88.7)	0.620	1	14 (11.3)	110 (88.7)	0.002	1 ^b^
Married/Living as a couple	33 (15.1)	186 (84.9)		1.39 (0.71–2.72)	8 (3.7)	211 (96.3)		0.30 (0.12–0.73)
Divorced/Separated/Widowed	8 (14.0)	49 (86.0)		1.28 (0.51–3.26)	0	57 (100)		NA
Parental status								
Yes	29 (13.2)	190 (86.8)	0.746	0.91 (0.51–1.61)	3 (1.4)	216 (98.6)	<0.001	0.12 (0.03–0.41) ^c^
No	26 (14.4)	155 (85.6)		1	19 (10.5)	162 (89.5)		1
Religious affiliation								
Yes	16 (15.4)	88 (84.6)	0.574	1.20 (0.64–2.25)	5 (4.8)	99 (95.2)	0.719	0.83 (0.30–2.31)
No	39 (13.2)	257 (86.8)		1	17 (5.7)	279 (94.3)		1
Economic status								
Very poor/Poor	15 (8.6)	159 (91.4)	0.009	1	11 (6.3)	163 (93.7)	0.527	1.32 (0.56–3.12)
Average/Rich	40 (17.7)	186 (82.3)		2.27 (1.22–4.35)	11 (4.9)	215 (95.1)		1
Source of HIV infection								
Sexual transmission	34 (15.2)	190 (84.8)	0.211	1	4 (1.8)	220 (98.2)	0.001	1
Injected drug use	11 (9.2)	108 (90.8)		0.57 (0.28–1.17)	13 (10.9)	106 (89.1)		6.75 (2.15–21.18)
Others	10 (17.5)	47 (82.5)		1.19 (0.55–2.58)	5 (8.8)	52 (91.2)		5.29 (1.37–20.38)

^a^OR _≥ High school vs Secondary school_ = 2.65, 95%CI 1.20–6.03

^b^OR _Single vs Married/Living as couple_ = 3.33, 95%CI 1.37–8.33

^c^Reversed OR = 8.33; 95%CI 2.44–33.33

**Table 2:** Bivariable association between health risk behaviours and clinical factors among Vietnamese HIV positive outpatients (*N* = 400)

Clinical factors	Unsafe sexual practice				Illicit drug use			
	Yes *n (%)*	No *n (%)*	*p*	OR (95% CI)	Yes *n (%)*	No *n (%)*	*p*	OR (95% CI)
General health status								
Poor/Fair	33 (14.5)	194 (85.5)	0.600	1.17 (0.65–2.08)	11 (4.8)	216 (95.2)	0.511	0.75 (0.32–1.77)
Very good/Good	22 (12.7)	151 (87.3)		1	11 (6.4)	162 (93.6)		1
Body Mass Index								
Under-weight	13 (10.7)	108 (89.3)	0.369	1	13 (10.7)	108 (89.3)	0.011	1
Normal	35 (14.4)	208 (85.6)		1.40 (0.71–2.75)	9 (3.7)	234 (96.3)		0.32 (0.13–0.77) ^a^
Overweight	7 (19.4)	29 (80.6)		2.01 (0.73–5.48)	0	36 (100)		NA
Time since HIV diagnosis in years								
Median (IQR)	5.3 (2.8–6.8)	5.8 (3.5–6.7)	0.143	0.91 (0.81–1.02)	4.3 (2.4–6)	5.8 (3.3–6.7)	0.101	0.87 (0.73–1.04)
Most recent CD4 cell count								
Median (IQR)	381 (282–642)	422 (274–582)	0.885	1.02 (0.91–1.14) ^b^	412.5 (210–567)	418.5 (279–587)	0.593	0.94 (0.79–1.13) ^b^
Time since ARV treatment in years								
Median (IQR)	3.80 (1.80–5.60)	4.22 (1.78–6.03)	0.448	0.95 (0.84–1.08)	2.0 (1.6–5.4)	4.24 (1.82–5.91)	0.080	0.82 (0.67–1.00)
Current HIV stage								
1	51 (13.9)	317 (86.1)	0.484	1	20 (5.4)	348 (94.6)	0.411	1
2	0	10 (100)		NA	0	10 (100)		NA
3	3 (17.6)	14 (82.4)		1.33 (0.37–4.80)	1 (5.9)	16 (94.1)		1.09 (0.14–8.62)
4	1 (20.0)	4 (80.0)		1.55 (0.17–14.18)	1 (20.0)	4 (80.0)		4.35 (0.46–40.75)
Presence of other chronic diseases								
Yes	13 (13.4)	84 (86.6)	0.909	0.96 (0.49–1.88)	5 (5.2)	92 (94.8)	0.864	0.91 (0.33–2.55)
No	42 (13.9)	261 (86.1)		1	17 (5.6)	286 (94.4)		1
History of delaying ARV treatment								
Yes	2 (8.3)	22 (91.7)	0.757	0.56 (0.13–2.45)	2 (8.3)	22 (91.7)	0.634	1.60 (0.35–7.29)
No	52 (14)	320 (86.0)		1	20 (5.4)	352 (94.6)		1
History of opportunistic disease diagnosis								
Yes	32 (12.5)	225 (87.5)	0.312	0.74 (0.42–1.33)	16 (6.2)	241 (93.8)	0.393	1.52 (0.58–3.96)
No	23 (16.1)	120 (83.9)		1	6 (4.2)	137 (95.8)		1
Adherence to HIV medications								
Good	49 (13.3)	319 (86.7)	0.036	1	20 (5.4)	348 (94.6)	0.180	1
Average	1 (5.6)	17 (94.4)		0.38 (0.05–2.94)	0	18 (100)		NA
Poor	5 (35.7)	9 (64.3)		3.62 (1.16–11.24)	2 (14.3)	12 (85.7)		2.90 (0.61–13.85)

^a^Reversed OR = 3.13; 95% CI 1.30–7.69

^b^For every 100 units change

**Table 3:** Bivariable association between health risk behaviours and psychosocial factors among Vietnamese HIV positive outpatients (*N* = 400)

Psychosocial and mental health factors	Unsafe sexual practices				Illicit drug use			
	Yes *n (%)*	No *n (%)*	*P*	OR (95% CI)	Yes *n (%)*	No *n (%)*	*p*	OR (95% CI)
Have family members diagnosed HIV positive								
Yes	25 (20.5)	97 (79.5)	0.009	2.13 (1.19–3.81)	3 (2.5)	119 (97.5)	0.096	0.34 (0.10–1.18)
No	30 (10.8)	248 (89.2)		1	19 (6.8)	259 (93.2)		1
HIV status disclosure								
Yes	50 (13.7)	314 (86.3)	0.999	0.99 (0.37–2.66)	21 (5.8)	343 (94.2)	0.708	2.14 (0.28–16.41)
No	5 (13.9)	31 (86.1)		1	1 (2.8)	35 (97.2)		1
Family support								
Yes	48 (13.3)	312 (86.7)	0.468	0.73 (0.30–1.73)	22 (6.1)	338 (93.9)	0.149	NA
No	7 (17.5)	33 (82.5)		1	0	40 (100)		
Social support								
Yes	19 (20.9)	72 (79.1)	0.025	2.00 (1.08–3.70)	7 (7.7)	84 (92.3)	0.297	1.63 (0.64–4.14)
No	36 (11.7)	273 (88.3)		1	15 (4.9)	294 (95.1)		1
Professional support								
Yes	27 (11.0)	219 (89.0)	0.042	0.55 (0.31–0.98) ^a^	14 (5.7)	232 (94.3)	0.832	1.10 (0.45–2.69)
No	28 (18.2)	126 (81.8)		1	8 (5.2)	146 (94.8)		1
Number of stressful life experiences in past 30 days								
0	7 (14.3)	42 (85.7)	0.439	1	2 (4.1)	47 (95.9)	0.929	1
1–2	18 (13.1)	119 (86.9)		0.91 (0.35–2.33)	8 (5.8)	129 (94.2)		1.46 (0.30–7.11)
3–4	16 (11.2)	127 (88.8)		0.76 (0.29–1.96)	7 (4.9)	136 (95.1)		1.21 (0.24–6.03)
5–6	11 (22.0)	39 (78.0)		1.69 (0.60–4.80)	4 (8.0)	46 (92.0)		2.04 (0.36–11.71)
> 6	3 (14.3)	18 (85.7)		1.00 (0.23–4.31)	1 (4.8)	20 (95.2)		1.17 (0.10–13.71)
Symptoms of alcohol use disorder (WHO-AUDIT) ^b^								
Yes	7 (13.2)	46 (86.8)	0.902	0.95 (0.40–2.22)	3 (5.7)	50 (94.3)	0.999	1.04 (0.30–3.63)
No	48 (13.8)	299 (86.2)		1	19 (5.5)	328 (94.5)		1
Symptoms of substance use disorder (DAST) ^b^								
Yes	2 (15.4)	11 (84.6)	0.696	1.15 (0.25–5.31)	10 (76.9)	3 (23.1)	<0.001	104.17 (25.37–427.71)
No	53 (13.7)	334 (86.3)		1	12 (3.1)	375 (96.9)		1
Anxiety symptoms (PVPS-A) ^b^								
Yes	7 (16.7)	35 (83.3)	0.562	1.29 (0.54–3.07)	2 (4.8)	40 (95.2)	0.999	0.85 (0.19–3.75)
No	48 (13.4)	310 (86.6)		1	20 (5.6)	338 (94.4)		1
Depressive symptoms (CES-D) ^b^								
Yes	19 (13.0)	127 (87.0)	0.746	0.91 (0.50–1.65)	9 (6.2)	137 (93.8)	0.659	1.22 (0.51–2.92)
No	36 (14.2)	218 (85.8)		1	13 (5.1)	241 (94.9)		1
Symptoms of HIV associated dementia (IHDS) ^b^								
Yes	22 (13.8)	137 (86.2)	0.967	1.01 (0.57–1.81)	8 (5.0)	151 (95.0)	0.738	0.86 (0.35–2.10)
No	33 (13.7)	208 (86.3)		1	14 (5.8)	227 (94.2)		1

^a^Reversed OR = 1.82; 95% CI 1.02–3.23. ^b^Yes = Above cutoff, No = Below cutoff

Illicit drug use was associated with being male (OR = 6.25, 95% CI 1.41–25.00), single (OR = 3.33, 95% CI 1.37–8.33), childless (OR = 8.33, 95% CI 2.44–33.33), having acquired HIV through injecting drugs (OR = 6.75, 95% CI 2.15–21.18) (Table 1), lower body mass index (BMI) (OR = 3.13, 95%CI 1.30–7.69) (Table 2) and having symptoms of SUD (OR = 104.17, 95% CI 25.37–427.71) (Table 3). However, no association was found between illicit drug use and symptoms of depression (OR = 1.22, 95% CI 0.51–2.92), anxiety (OR = 0.85, 95% CI 0.19–3.75), AUD (OR = 1.04, 95% CI 0.30–3.63) or HAD (OR = 0.86, 95% CI 0.35–2.10).

### Multivariable analysis

**Fig. 1:**
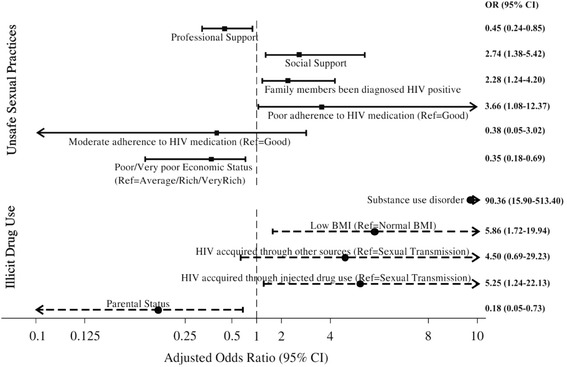
Multivariable correlates of high-risk health behaviours among Vietnamese HIV positive outpatients

**Fig. 2:**
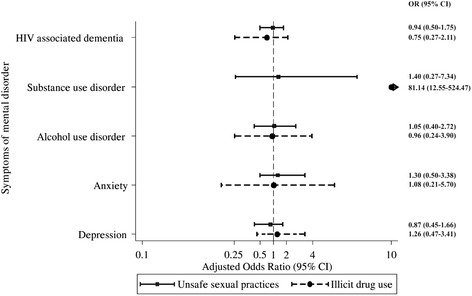
Adjusted association between symptoms of mental disorders and high-risk health behaviours among Vietnamese HIV positive outpatients. Note: For illicit drug use, adjusting variables include sex, age, parental status, source of HIV infection, Body Mass Index and symptoms of substance use disorder. For unsafe sexual practices, adjusting variables include sex, age, economic status, adherence to HIV treatment, having family member diagnosed HIV positive, receiving social support, receiving professional support

Initial multivariable modelling identified five potential demographic, clinical and psychosocial multivariable predictors of the USP measure of HRB. This initial modelling found a lower likelihood of USP among participants with poor economic status (OR = 0.35, 95% CI 0.18–0.69) and those who had received professional support (OR = 0.45, 95% CI 0.24–0.85). In contrast, HIV positive outpatients were more likely to have USP when they reported receiving social support (OR = 2.74, 95% CI 1.38–5.42), had a family member who had been diagnosed HIV positive (OR = 2.28, 95% CI 1.24–4.20) and had poor adherence to HIV medication (OR = 3.66, 95% CI 1.08–12.37) (Fig. 1). The final multivariable model (Fig. 2) used these five variables as well as age and sex as control variables in a model predicting USP from SOMD. In this model, none of the measures of SOMD were associated with USP. USP was not associated with symptoms of depression (OR = 0.87, 95% CI 0.45–1.66), anxiety (OR = 1.30, 95% CI 0.50–3.38), AUD (OR = 1.05, 95% CI 0.40–2.72), SUD (OR = 1.40, 95% CI 0.27–7.34) or HAD (OR = 0.94, 95% CI 0.50–1.75).

Initial multivariable modelling identified four potential demographic, clinical and psychosocial multivariable predictors of illicit drug use. Participants who had children were less likely to report illicit drug use (OR = 0.18; 95% CI 0.05–0.73). However, illicit drug use was positively associated with having low BMI (OR = 5.86; 95% CI 1.72–19.94), HIV acquired through injecting drugs (OR = 5.25; 95% CI 1.24–22.13) and having symptoms of SUD (OR = 90.36; 95% CI 15.90–513.40) (Fig. 1). The final multivariable model (Fig. 2) used these four variables with the addition of sex and age as control variables in a model predicting illicit drug use from SOMD. Illicit drug use was not associated with symptoms of depression (OR = 1.26; 95% CI 0.47–3.41), anxiety (OR = 1.08; 95%CI 0.21–5.70), AUD (OR = 0.96, 95%CI 0.24–3.90) or HAD (OR = 0.75, 95%CI 0.27–2.11). However, participants who had symptoms of SUD were more likely to report illicit drug use (OR = 81.14; 95% CI 12.55–524.47).

## Discussion

This study is one of a limited number that has investigated both SOMD and HRB and the relationship between the two in a large sample of Vietnamese HIV positive outpatients. While high prevalences of SOMD such as symptoms of depression (36.5%) and of HAD (39.8%) were identified in our sample, and this is consistent with previous research conducted in Vietnam and other countries [30, 31, 45, 48, 49], neither symptoms of depression nor of HAD were associated with USP or illicit drug use. Similarly, AUD and anxiety symptoms did not significantly predict USP or illicit drug use. Of the symptom measures used here, only the DAST, included to measure symptoms of SUD, was a significant predictor of illicit drug use.

The high prevalence of symptoms of depression and of HAD identified in our sample is consistent with previous research conducted in Vietnam where 40% of PLHIV in a northern province and 32% of HIV positive outpatients in HCMC were reported as having depression [30, 31]. The HAD prevalence indicated by the IHDS in the current study was higher than the prevalence of 11% identified by psychiatrist’s interview in Vietnam [31] but was similar to prevalences reported in previous studies using the IHDS in the United States of America (37.9%), Canada (39.4%), and India (32.5%) [45, 48, 49], although lower than most studies in Africa, where reported HAD prevalence has ranged from 54.4 to 85% [50, 51]. Since these studies also employed the IHDS the difference in HAD prevalence might be due to HIV severity, especially at ART initiation [50]. Our findings indicate an urgent need for screening for SOMD and provision of appropriate support and treatment for HIV positive outpatients in Vietnam.

The prevalence of USP among HIV positive outpatient in this study (13.8%) was lower than the prevalence reported by Thanh et al. [2] among PLHIV in community and commune health clinics (20%) [2]. It is also lower than the prevalence of approximately 50% among PLHIV in St Petersburg, Russia [3] and among PLHIV in Pennsylvania, USA [5] and the 34% reported among PLHIV in Las Vegas Valley, USA [7]. Many Vietnamese PLHIV hold the incorrect belief that sexual activity weakens the body and that reducing or avoiding sexual activity can protect one’s health [22] and so it is possible that the HIV positive outpatients in the present study have chosen to reduce or avoid sexual activity in order to improve their health. Another possible factor that might explain this difference was the lower prevalence of symptoms of depression found in the earlier studies, since a qualitative study among Vietnamese PLHIV found that depression can reduce sexual desire, pleasure and frequency of sexual activities [22].

In our study only 5.5% of participants reported illicit drug use during the past 12 months while Thanh et al. [2] reported injecting drug use during the past month to be 51.6% in a Vietnamese sample of PLHIV [2]. This discrepancy between findings is perhaps surprising, particularly given that we assessed any illicit drug use, including, but not limited to, injected substances, while Thanh et al. assessed injecting drug use specifically. It would be expected that a higher proportion of PLHIV would engage in illicit drug use compared to injecting drug use only. While it is difficult to reconcile the differences in findings across the two studies, and further research will need to examine drug use in future research, we suggest that one factor may be the difference between the samples recruited in the two studies. While the majority of Thanh et al’s sample were newly-diagnosed HIV positive patients, with 54% of participants diagnosed within 1 year of HIV diagnosis [2], our study was carried out in outpatient clinics where participants had an average of 5.7 years since HIV diagnosis and most of them (99.0%) had been taking ART for an average of 4.2 years. Across Vietnam, only 46.9% of PLHIV receive ART [52]. To be eligible for ART, PLHIV must complete several health education and counselling workshops about HIV, ART, adherence and positive living [53]. Therefore, it is possible that the HIV positive outpatients in our study who were on ART and had attended these programs were more aware of HIV related risk behaviours, such as illicit drug use, and how these negatively impact on quality of life and thus may have reduced their drug use behaviour. In contrast, participants in Thanh et al.’s [2] study were recently diagnosed with HIV and took part in the study prior to receiving counselling and education. Consistent with this, low prevalence rates for injecting drug use, heroin, use and ecstasy, amphetamine, and cocaine use have been reported among French HIV positive outpatients who had been on ART for an average of 6.4 years [54]. Survey data collected from 15 OPCs in HCMC in 2015 where 92% of HIV positive outpatients were on ART also found reported illicit drug use to be low [55]. Although the prevalence of illicit drug use among PLHIV was not available, a report from the Asia-Pacific region indicated prevalence of unsafe injection practices among PLHIV varied across the countries, ranging from 2% in Malaysia to 75% in Philippines [56].

The factors associated with USP in this study were consistent with previous research where HIV positive outpatients with family members diagnosed HIV positive and poor adherence to HIV medication were more likely to report USP [3, 5] and clinical indicators such as CD4 cell count, opportunistic diseases or any other comorbidity were not good predictors of USP [2, 5]. Given these non-significant associations we suggest that populations targeted for HRB interventions at HIV outpatient clinics should not be selected based on clinical factors alone. Targeting sub-populations for intervention should also take into account economic status since the current study demonstrated that HIV positive outpatients who perceived their economic status as poor or very poor were less likely to have USP compared to those with average or high economic status. Interestingly, we found that HIV positive outpatients who reported receiving social support had high odds of reporting USP, and those who reported professional support were less likely to report USP. However, such relationships might be affected by the complex nature of social support which warrants further investigation [57].

Similar to other studies, we found that HIV positive outpatients who had low BMI or acquired HIV through injecting drugs, as well as those with symptoms of SUD, were more likely to report illicit drug use. The link in our findings between SUD as a SOMD and illicit drug use as a HRB is not surprising, given the probable role of illicit drug use in substance abuse disorder. In contrast, HIV positive outpatients who reported that they were parents were less likely to use illicit drugs. Illicit drug use can complicate HIV treatment, reduce treatment adherence and reduce viral suppression [58]. Those who reported they had acquired HIV through injecting drug use or other substance use specifically may no longer use drugs, if their symptoms of SUD have been treated. Thus, PLHIV should be routinely screened for drug use related disorders and interventions to reduce HRB should target those with symptoms of SUD. Such routine screening and intervention during clinical care has been shown to be practical and effective in reducing HRB among PLHIV, as well as optimizing HIV treatment and improving PLHIV’s quality of life [52].

Despite the view that there is an association between SOMD and HRB [20, 21], the current study demonstrated that HIV positive outpatients who had SOMD did not have higher odds of HRB either with or without adjusting for associated factors of HRB. Therefore, interventions for HRB should not be solely based on SOMD, except for illicit drug use among those who had symptoms of SUD. This finding supports the results of a meta-analysis of 34 studies from countries outside Vietnam where SOMD such as depression and anxiety were not significantly associated with USP [18]. One possibility as suggested by Crepaz et al. [18] was the incomplete overlap periods for the measures of SOMD and HRB. In our study, the SOMD scales limited the symptoms to short periods, normally 1 week, while HRB were measured for a 12-month period. As such, the temporal disconnection of these measures might limit the strength of the statistical association. However, HRB measurement within a short timeframe such as 1 week and SOMD in a longer period (i.e. 12 months) also has limitations such as measurement bias. Additionally, our study did not assess for symptoms of psychosis or mania, two conditions that could have resulted in high rates of USP or illicit drug use. Finally, it may be that the relationship between SOMD and HRB in PLHIV depends on the stage of the illness and the characteristics of the sample. As noted above, our sample had completed health education and counselling workshops about HIV, ART, adherence and positive living, were receiving ART and had been living with HIV for many years.

The study had several limitations. The cross-sectional design limits explanations about the causal relationship between SOMD or other factors and HRB. Further studies using prospective cohort study designs, where SOMD and HRB are observed from ART initiation and followed up over time, are needed to clarify the causal pathway between SOMD and HRB. Despite the availability of SOMD and HRB validated screening scales, the incomplete overlap of measurement periods as discussed earlier should be taken into account in future studies. The study estimated HRB through self-report and thus participants might have experienced recall bias or may have been unwilling to accurately report these behaviours for a range of reasons including concerns about social stigma. As such, the rate of HRB found in this study might be an under-estimate. The low number of participants reporting HRB may also have limited us from finding associations between SOMD and HRB. Further research is also needed to evaluate different modes of assessing risky behaviours among Vietnamese PLHIV so that estimates can be comparable across studies and less susceptible to bias. Lastly, the study was conducted in one of the largest cities in Vietnam among HIV positive outpatients, almost all of whom were on ART. This may limit the applicability of the study findings to other populations of PLHIV in Vietnam, including PLHIV living in rural areas, PLHIV who are not on ART and those who are under care and treatment in hospital settings. Additionally, due to resource constraints, our sample was recruited from only two of the 30 OPCs in HCMC. While the two clinics were randomly selected and each of the 30 clinics have the same admission criteria and services for HIV positive outpatients, it is possible that there may be some differences in the sample characteristics of the HIV positive outpatients at these two clinics compared to the other OPCs. Further research in other settings in Vietnam, both in HCMC and in areas of Vietnam outside HCMC is needed.

## Conclusions

SOMD were very common among Vietnamese HIV positive outpatients however there was no association between SOMD and HRB except between symptoms of SUD and illicit drug use. Given the prevalence of SOMD identified it is likely that early screening for SOMD would lead to appropriate referral for further SOMD assessment and management. This is likely to be beneficial since early treatment of SOMD is generally more effective and provides HIV positive outpatients with better health and quality of life. Unsafe sexual practices and illicit drug use were reported by 13.8 and 5.5% of participants respectively. Further research examining the factors that predict these behaviours and the development of interventions to reduce the prevalence of HRB is crucial. Routine HIV prevention counselling during clinical care targeting HIV positive outpatients with the risk factors for USP and illicit drug use could be beneficial for patient’s health, quality of life and for prevention of secondary transmission of HIV or infection with other sexually transmitted diseases.

## Abbreviations

ART: Anti-Retroviral Therapy
BMI: Body mass index
CES-D: Center for Epidemiologic Studies – Depression scale
DAST: Drug Abuse Screening Test
HAD: HIV associated dementia
HCMC: Ho Chi Minh City
HRB: Health risk behaviours
IHDS: International HIV Dementia Scale
OPC: Outpatient clinic
PLHIV: People living with HIV/AIDS
PVPS-A: Phan Vietnamese Psychiatric Scale-Anxiety Scale
SOMD: Symptoms of mental disorders
USP: Unsafe sexual practices
WHO-AUDIT: World Health Organization Alcohol Use Disorder Identification Test
